# Elevated Stress-Hemoconcentration in Major Depression Is Normalized by Antidepressant Treatment: Secondary Analysis from a Randomized, Double-Blind Clinical Trial and Relevance to Cardiovascular Disease Risk

**DOI:** 10.1371/journal.pone.0002350

**Published:** 2008-07-16

**Authors:** Ma-Li Wong, Chuanhui Dong, Karin Esposito, Sarika Thakur, Weiqing Liu, Robert M. Elashoff, Julio Licinio

**Affiliations:** 1 Department of Psychiatry and Behavioral Sciences, Leonard M. Miller School of Medicine at the University of Miami, Miami, Florida, United States of America; 2 Department of Psychiatry and Biobehavioral Sciences, David Geffen School of Medicine at University of California Los Angeles, Los Angeles, California, United States of America; 3 Department of Biomathematics, University of California Los Angeles, Los Angeles, California, United States of America; James Cook University, Australia

## Abstract

**Background:**

Major depressive disorder (MDD) is an independent risk factor for cardiovascular disease (CVD); the presence of MDD symptoms in patients with CVD is associated with a higher incidence of cardiac complications following acute myocardial infarction (MI). Stress-hemoconcentration, a result of psychological stress that might be a risk factor for the pathogenesis of CVD, has been studied in stress-challenge paradigms but has not been systematically studied in MDD.

**Methods:**

Secondary analysis of stress hemoconcentration was performed on data from controls and subjects with mild to moderate MDD participating in an ongoing pharmacogenetic study of antidepressant treatment response to desipramine or fluoxetine. Hematologic and hemorheologic measures of stress-hemoconcentration included blood cell counts, hematocrit, hemoglobin, total serum protein, and albumin, and whole blood viscosity.

**Findings:**

Subjects with mild to moderate MDD had significantly increased hemorheologic measures of stress-hemoconcentration and blood viscosity when compared to controls; these measures were correlated with depression severity. Measures of stress-hemoconcentration improved significantly after 8 weeks of antidepressant treatment. Improvements in white blood cell count, red blood cell measures and plasma volume were correlated with decreased severity of depression.

**Conclusions:**

Our secondary data analyses support that stress-hemoconcentration, possibly caused by decrements in plasma volume during psychological stress, is present in Mexican-American subjects with mild to moderate MDD at non-challenged baseline conditions. We also found that after antidepressant treatment hemorheologic measures of stress-hemoconcentration are improved and are correlated with improvement of depressive symptoms. These findings suggest that antidepressant treatment may have a positive impact in CVD by ameliorating increased blood viscosity. Physicians should be aware of the potential impact of measures of hemoconcentration and consider the implications for cardiovascular risk in depressed patients.

## Introduction

Cardiovascular disease (CVD) risk has been linked to several emotional and psychological factors, including stress and depression. Mental stress can elicit acute coronary events and is considered a risk factor for CVD [1]. Depressive symptoms, which are common among patients with ischemic heart disease and those recovering from an acute myocardial infarction (MI) [2], [3] are associated with an increased risk of CVD, MI, and cardiac mortality [3]–[7] In patients with CVD, the presence of major depressive disorder (MDD) is associated with higher rates of cardiac complications (such as reinfarction and the need for revascularization) [3] and a two-to-four times increased risk of cardiac mortality compared with non-depressed patients [4]–[6]. Recently, the INTERHEART study reported an increase risk of acute MI across a variety of psychosocial stressors, including depression, in a large, multinational, case-control study [8]. Given that the World Health Organization Global Burden of Disease Survey estimates that by the year 2020, coronary heart disease and depression will be the first and second most disabling conditions worldwide, respectively, understanding the relationships between these disorders is critical [9].

A number of mechanisms underlying the link between stress, depression and cardiovascular disease have been proposed (for a comprehensive review, see reference [10]). Stress-related changes in sympathetically mediated hemorheologic factors related to blood viscosity and hemoconcentration, such as hematocrit and total plasma protein, may provide a link between behavioral stress and the development of CVD [11]. Increased blood viscosity has been associated with cardiac ischemia, myocardial infarction and necrosis, and stroke [12]–[14]. Hemoconcentration increases the risk of ischemia and thrombosis [15], [16], and increased levels of hematocrit and hemoglobin have been identified as independent risk factors for CVD [17].

Behavioral/emotional stress and chronic anxiety have been shown to cause changes in hemorheologic measures, possibly due to increases in catecholamines and blood pressure [18]. These changes have been referred to variably as stress-hemoconcentration, stress polycythemia, relative polycythemia, pseudopolycythemia, or spurious polycythemia and are characterized by an increased red-cell-mass-to-plasma ratio resulting from a reduction in plasma volume in the presence of normal red cell counts. Shifts of fluids out of the blood and into other compartments of the body are responsible for this manifestation of hemoconcentration [18]. Several studies have documented hemoconcentration in response to acute mental or psychomotor challenges. Maes *et al.* studied students under two baseline conditions, a few weeks before and after a difficult exam, and the day before the stressor, and reported significant stress-induced hematological changes [19]. Others have looked at more acute stress tasks (3–20 minutes) in the laboratory and report changes consistent with hemoconcentration [20]–[22]. Such findings have implications for cardiovascular disease risk in acute and chronic stress.

Despite the importance of the hemorheologic changes described in stress-hemoconcentration and the overlap between stress and depression in studies of cardiovascular disease risk, such changes have not been systematically studied in MDD. The present study is a secondary analysis of data from a randomized, double-blind trial of fluoxetine versus desipramine in a group of outpatients with mild to moderate MDD before and after antidepressant treatment compared to a group of ethnically-matched controls. The primary analysis focused on genetic markers of depression and antidepressant treatment response and has been reported elsewhere [23] Our hypothesis for the secondary analysis presented in this paper was that subjects with MDD would exhibit hematological and hemorheologic measures compatible with stress-hemoconcentration, and that these measures would improve with successful antidepressant treatment.

## Methods

### Study population

This study was approved by the University of California, Los Angeles (UCLA) and University of Miami (UM) IRBs and has been registered in the public database clinicaltrials.gov (NCT00265291). The study population consisted of control and MDD individuals. All subjects gave written informed consent and received comprehensive psychiatric and medical assessment. We used diagnostic and ratings instruments that have been fully validated in English and in Spanish, and conducted all assessments in the subject's primary language.

We studied 146 outpatient depressed subjects, all of whom were Mexican-Americans (defined as having at least 3 grandparents born in Mexico) aged 19–65 years, who were participating in an ongoing randomized, double-blind pharmacogenetic study of antidepressant response to desipramine or fluoxetine and completed the 8-week treatment trial (see Table 1 for population characteristics). All depressed subjects had a current episode of unipolar major depression as diagnosed by the Structured Clinical Interview for DSM-IV (SCID). Severity of depression was assessed with the 21-Item Hamilton Depression Rating Scale (HAM-D21) [24]; a score of 18 or greater, with item number 1 (depressed mood) rated 2 or greater, was required for inclusion. The SCID and HAM-D21 have been validated in English and Spanish, and all assessments were conducted in the subject's primary language. Exclusion criteria included any primary Axis I disorder other than MDD (e.g. dementia, psychotic illness, bipolar disorder, adjustment disorder); electroconvulsive therapy in the last 6 months; previous lack of response to desipramine or fluoxetine; current, active suicidal ideation with a plan and strong intent; or any other antidepressant treatment within the 2 weeks prior to enrollment. Patients enrolled in this protocol were either drug-naïve or drug-free for at least two weeks; in that case, their antidepressant medication had been discontinued for clinical reasons or because of non-adherence. Subjects with any active medical illnesses that could be etiologically related to the ongoing depressive episode (e.g. untreated hypothyroidism, cardiovascular accident within the past 6 months, uncontrolled hypertension or diabetes), and who were pregnant, lactating, currently using medications with significant central nervous system activity (e.g. benzodiazepines), exhibiting illicit drug use and/or alcohol abuse in the last 3 months, or currently enrolled in psychotherapy were also excluded. Female patients were required to use contraception during our treatment trial, but only 4 used hormonal contraceptive agents. Our patients were predominantly non-smokers (only 6 were smokers), and 37 patients were taking other medications during our trial.

**Table 1:** Demographic Data and BMI for Controls and MDD Subjects

Subject*	Gender†	N (%)	Variable‡	Mean±SD
Controls	Female	33 (72)	Age	35.9±8.9
			BMI	28.6±4.5
	Male	13 (28)	Age	33.2±7.7
			BMI	29.4±4.6
	All	46	Age	35.2±8.6
			BMI	28.8±4.5
MDD Subjects	Female	97 (66)	Age	36.4±9.7
			BMI	28.3±5.6
	Male	49 (34)	Age	38.9±9.5
			BMI	28.2±4.0
	All	146	Age	37.2±9.7
			BMI	28.3±5.1

* p for unpaired t-test is 0.194 for age and 0.543 for BMI between controls and MDD patients.

† p for Chi-square test is 0.503 for gender ratio between controls and MDD patients.

‡ Age in year; BMI: body mass index defined as weight in kilogram divided by the square of height in meters.

We studied 46 ethnically-matched control subjects (Table 1) who were recruited from the same Mexican-American Los Angeles community and evaluated by the same bilingual, clinical research team at the Center for Pharmacogenomics and Clinical Pharmacology, David Geffen School of Medicine at UCLA [25]. Control subjects were in good general health and free of ongoing physical illness. They showed no evidence of major psychiatric illness in clinical and structured interview. Control and MDD individuals received the same comprehensive psychiatric and medical assessments.

### Antidepressant treatment

All MDD subjects reported here completed 8 weeks of a randomized, double-blind trial of antidepressant treatment response to desipramine or fluoxetine as part of a pharmacogenetic study. The treatment had two phases. Phase 1 was a 1-week, single-blind placebo lead-in phase to eliminate placebo responders. Subjects who continued to meet the inclusion criteria after Phase 1 were randomly assigned to one of two treatment groups in a double-blind manner in Phase 2 during which they received fluoxetine 10–40 mg/day or desipramine 50–200 mg/day, for 8 weeks, with a dose escalation based on clinical outcomes. All subjects had 9 weeks of structured follow-up assessments. Our primary clinical outcome measure within the depressed group receiving antidepressant treatment was the HAM-D21. Remitter was defined as the patients who had a final HAM-D21 score <8.

### Hemorheologic measures

Blood was collected from a vein in the antecubital fossa before beginning antidepressant treatment (week −2) and at the end of treatment (week 8). All samples were drawn after the subjects had rested in a supine position for 5–10 minutes. Samples for white blood cell (WBC) and red blood cell (RBC) count, hematocrit (HCT) and hemoglobin (HGB) levels were drawn into 7 ml ethylenediaminetetraacetic acid (K_2_ EDTA) BD-Vacutainer^®^ tubes. Total serum protein (TSP) and albumin samples were drawn into chilled 7-ml K_2_ EDTA-treated BD-Vacutainer^®^ tubes and placed on ice until centrifuged at 3000 rpm for 10 minutes at 4°C. Blood count and protein analysis were performed by the UCLA Clinical Laboratories and Pathology Services using a Sysmex XE-2100 (Sysmex Co, Kobe, Japan) and Synchron LX^®^20 (Beckman Coulter, Fullerton, CA), respectively.

### Whole Blood Viscosity (WBV) Estimation

WBV was determined in centipoises (cP) at a shear rate of 208 seconds^−1^ using the following equation: WBV = 0.12×HCT (%)+0.17×serum proteins (g/dL)

This equation has been validated by de Simone *et al* [26]. By comparison with direct WBV measurements (*r* = 0.92, n = 50) in adults.

### Plasma Volume Estimation

Estimates in plasma volume changes before and after 8-weeks antidepressant treatment was calculated using the following formula:
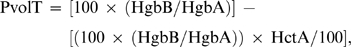
where PvolT is the plasma volume after antidepressant treatment, HgbB is the hemoglobin before treatment (at −2 week), HgbA and HctA are the hemoglobin and hematocrit after 8-week treatment, respectively [27].

### Statistical Analyses

Variables used for secondary data analyses included HAM-D21 scores and the following measurements: RBC, HGB, HCT, WBC, TSP, albumin levels and estimation of WBV. MDD subjects were analyzed before (week −2) and after treatment (week 8), and control subjects were measured at one time point for comparison.

To assess similarity between depressed and control subjects, unpaired t-tests were used to compare age and BMI means and a χ^2^ test was used to compare gender ratios. To examine the potential effects of gender and age, unpaired t-tests were also performed for the difference in blood measurements between female and male, and Pearson correlation analysis was conducted for the correlation of age with blood measurements. To adjust for gender and age, a general linear model (GLM) was employed to conduct multivariate tests (MANOVA) to compare blood measurements between MDD subjects and controls or between desipramine- and fluoxetine- treated MDD subjects. Paired T-tests were used to compare blood measurements before and after treatment in MDD subjects. Spearman partial correlation was used to measure the degree of association of HAM-D21 score with the blood measurements by controlling for age and gender. Stepwise logistic regression analysis was conducted to screen the predictors that allow a differentiation between remitter and non-remitter patients using the baseline HAM-D21 score, baseline blood measurements, antidepressant medication, age, and gender variables.

All analyses were performed using SPSS version 14.0 (SPSS Inc., Chicago, IL, USA), except for Spearman partial correlation analysis which was conducted with SAS version 9.1 (SAS Institute Inc., Cary, NC, USA). As HAM-D21 score is an ordinal measurement rated on a 5-point scale for each item (from “0 – not present” to “4 – severe”) and did not follow normal distribution when used as an interval variable, Spearman partial correlation was used. A significance level of 0.05 was used for all statistical testing, and the Bonferroni post-hoc method of correction was used to correct for multiple testing.

### Role of Funding Sources:

This study was funded by the National Institute of General Medical Sciences (NIGMS), the National Institute of Diabetes and Digestive and Kidney Diseases (NIDDK), and the National Center for Research Resources (NCRR). The sponsors were not involved in the study design; the collection analysis and interpretation of data; the writing of the report; or the decision to publish.

## Results

### Hemorheologic and Hematological Measures in MDD Subjects and Controls

Our data are compatible with classical findings that gender influences red blood cell (RBC) measurements and albumin levels. RBC measurements are correlated with gender; female subjects had lower RBC count (*p*<0.0001; 4.5±0.3×10^6^/ µL in females vs 5.1±0.4×10^6^/ µL in males), lower HGB levels (*p*<0.0001; 13.2±1.1 g/dL in females vs 15.3±1.0 g/dL in males) and lower albumin levels (*p* = 0.0004; 4.1±0.3 g/dL in females vs 4.3 g/dL±0.3 g/dL in males). The results of correlation analysis showed that age was conversely correlated with TSP (r = −0.17, *p* = 0.019) and albumin (r = −0.18, *p* = 0.012).

Therefore, we performed multivariate analysis of variance (MANOVA) to compare group differences in hemorheologic measures and WBC count by controlling for gender and age (Table 2). Our results showed that before treatment, MDD subjects had significantly increased hemorheologic measures in total RBC count (*p* = 0.048), HBG (*p* = 0.005), HCT (*p* = 0.001), TSP concentrations (*p* = 0.010), and total WBC count (*p* = 0.039). Albumin levels showed a trend towards an increase when compared to controls (*p* = 0.052). MDD subjects also had higher estimated WBV than controls (*p*<0.001). After treatment, no significant differences in the hemorheologic measures or WBC count were found between MDD subjects and controls (all *p* values≥0.088).

**Table 2:** Hemorheologic Measures and WBC Count in MDD Subjects before Treatment (week −2) and Controls

Hemorheologic Measures	Controls (N = 46)*	MDD Subjects (N = 146)*	*p* †
RBC count −10^6^/µL	4.6±0.5	4.7±0.4	**0.048**
HGB – g/dL	13.4±1.6	14.0±1.4	**0.005**
HCT −%	39.5±4.2	41.4±3.5	**0.001**
TSP – g/dL	7.1±0.5	7.3±0.5	**0.010**
Albumin – g/dL	4.1±0.4	4.2±0.3	0.052
WBV – cP	5.9±0.5	6.2±0.4	**<0.001**
WBC count −10^6^/µL	6.8±1.5	7.5±2.1	0.052

* Values are means±SD.

† p was based on MANOVA using GLM model by adjusting for age and gender.

### Hemorheologic Measures in MDD Subjects Before and After Antidepressant Treatment

In MDD subjects, hemorheologic parameters of stress-hemoconcentration improved after 8 weeks of antidepressant treatment (Table 3). Paired t-test analyses showed that all the six hemorheologic measures and the WBC count in MDD subjects decreased significantly after antidepressant treatment (*p*≤0.0003).

**Table 3:** Hemorheologic Measures and WBC Count in MDD Subjects before (week −2) and after (week 8) Treatment

Hemorheologic Measures	Number of Patients*	Before Treatment#	After Treatment#	*p* †
RBC count −10^6^/µL	146	4.7±0.4	4.6±0.4	**0.0003**
HGB – g/dL	146	14.0±1.4	13.8±1.4	**<0.0001**
HCT −%	146	41.4±3.5	40.6±3.6	**<0.0001**
TSP – g/dL	146	7.3±0.5	7.1±0.4	**<0.0001**
Albumin – g/dL	146	4.2±0.3	4.0±0.3	**<0.0001**
WBV – cP	146	6.2±0.4	6.1±0.5	**<0.0001**
WBC count −10^6^/µL	146	7.5±2.1	6.7±1.7	**<0.0001**

* Subjects with no missing data.

# Values are means±SD.

† p was based on paired t-test.

### Hemorheologic Measures in Desipramine- and Fluoxetine- Treated MDD Subjects

Measures of stress-hemoconcentration were not significantly different in subjects treated with desipramine or fluoxetine in MANOVA analysis. Our analyses showed that total RBC count, HBG, HCT, total WBC, TSP and albumin did not differ between the two groups in the final week of treatment.

### Hemorheologic Measures and HAM-D21 Score in MDD Subjects

Table 4 presents the results of Spearman partial correlation analyses by controlling for age and gender. Our data showed that before treatment, hemorheologic measures were correlated with HAM-D21 score with a correlation coefficient of 0.21 for WBC count (*p* = 0.01), 0.18 for RBC count (*p* = 0.027), 0.24 for HCT (*p* = 0.003), 0.29 for WBV (*p*<0.001), and 0.22 for albumin level (*p* = 0.009). Our data also showed that improvements in WBC count, RBC measures, and plasma volume percentage were positively correlated with the improvement in HAM-D21 score, although no statistical significant difference was found for the change in hemorheologic measures between remitters and non-remitters.

**Table 4:** Correlations of HAM-D21 Score, Hemorheologic Measures and WBC Count in MDD Subjects (N = 146)

Hemorheologic Measures before Treatment	HAM-D21 Score before Treatment		Change in Hemorheologic Measures with treatment	Change in HAM-D21 Score	
	*r*	*p* †		*r*	*p* †
RBC count −10^6^/µL	0.18	**0.027**	RBC count −10^6^/µL	0.22	**0.008**
HGB – g/dL	0.14	0.105	HGB – g/dL	0.18	**0.027**
HCT −%	0.24	**0.003**	HCT −%	0.22	**0.008**
TSP – g/dL	0.26	**0.002**	TSP – g/dL	0.14	0.086
Albumin – g/dL	0.22	**0.009**	Albumin – g/dL	0.13	0.115
WBV – cP	0.29	**<0.001**	WBV – cP	0.21	**0.010**
			Plasma volume¶	0.20	**0.017**
WBC count −10^6^/µL	0.21	0.010	WBC count −10^6^/µL	0.23	0.007

† p was based on Spearman partial correlation by controlling for age and gender.

¶ Plasma volume change % after treatment based on the method provided by Dill and Costill.^27^

### Baseline Hemorheologic Measures and Clinical Remission Status

Stepwise logistic regression analysis revealed that the significant predictors of remission included HAM-D21 score (OR = 0.85, 95%CI = 0.77–0.93, *p* = 0.0003), TSP (OR = 2.81, 95%CI = 1.03–7.67, *p* = 0.044) and albumin (OR = 0.13, 95%CI = 0.02–0.67, *p* = 0.015) at baseline. Taking together, the model yielded a 73% concordance between the observed and predicted remission status.

## Discussion

Our secondary data analyses indicate that hemorheologic measures of stress-hemoconcentration are present in Mexican-American individuals with mild to moderate MDD and that these measures decrease significantly after 8 weeks of antidepressant treatment to levels which were the same as those of controls. Measures we obtained in depressed subjects at rest (in the absence of any deliberate psychological challenge) are comparable to those elicited after a stressful challenge in other studies [19]–[22]. We propose that stress-hemoconcentration may contribute to the increased risk of CVD found in MDD.

Psychological stress has been identified as a risk factor for CVD, as a trigger of acute coronary events, and as a contributor to the pathogenesis of atherosclerosis and hypertension [10]. Thus, given our findings of stress-hemoconcentration at rest in MDD, this mechanism is also a potential link in the association of MDD and CVD. In MDD, stress-hemoconcentration may increase blood viscosity; this may lead to a decrease in pressure at vulnerable branching sites of coronary arteries and increased exposure time to atherogenic substances [18].

During stress, hemoconcentration can be at least partially explained by sympathetic nervous system activation (for a comprehensive review see reference [22]. Infusion of catecholamines has been shown to result in reductions of plasma volume with parallel rises in hematocrit, possibly due to increases in systemic pressure and capillary hydrostatic pressure that reflects passive movement of fluid out of the capillaries. Elevations in plasma catecholamines have also been described in MDD [28] and thus, sympathetic nervous system activation would also be a valid explanation for alterations of stress-hemoconcentration measures in MDD, but in this study we have not obtained concomitant measures of plasma catecholamines; therefore, this possible relationship remains to be directly documented.

Stress hemoconcentration has not been systematically studied in MDD. Several studies on relatively small numbers of depressed subjects (30–50) examining other systemic aspects of depression – inflammation and the acute phase response – have reported on some measures that overlap with those we examined. Total serum protein [29], albumin [30], as well as RBC, HGB and HCT [31] have been reported as decreased in depression, unlike the increase we report in our population. Several major differences exist between these studies and ours, the most significant being that the studies were all conducted on smaller, acute inpatient populations. Multiple factors may differentiate inpatients with depression from outpatients: higher severity of illness, with the possibility of decreased mobility and poor nutritional status, and greater duration of illness with more exposure to medication. We examined several potential confounds in our study and found no difference in our results if we eliminated individuals with history of cardiovascular problems (n = 8) or family history of cardiac problems (n = 17) or individuals taking other medication (n = 37) or using hormonal contraceptive agents (n = 4) or smoking (n = 6). One limitation of the current study is that we did not directly measure fluid and food intake; another is that we did not collect the information on smoking habits for all the patients and controls. No significant weight change was observed over the course of the study, and while the appetite item on the Beck Depression Inventory did show improvement, there was no significant correlation between appetite score change and change in hemoconcentration measures. A strength of our study is the focus on one ethnic group with strict inclusion criteria (3 of 4 grandparents born in Mexico), thus minimizing variation that could be introduced by a genetically more heterogenous population. It is unknown whether other ethnic groups could react differently.

**Figure 1:**
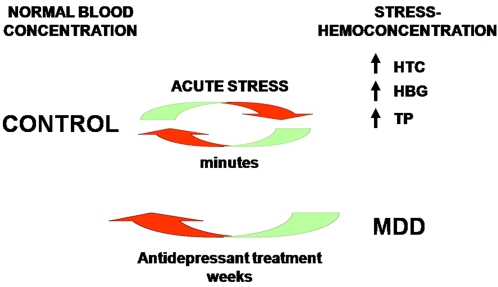
Relationship between acute stress, stress-hemoconcentration and MDD. Normal individuals can display hemorheologic measures of stress-hemoconcentration after acute psychological stress. Those measures return to baseline levels within minutes after the stress situation is terminated. Subjects with major depressive disorder (MDD) display stress-hemoconcentration at baseline, non-stressed conditions and those measures return to baseline levels in responders to an 8-week treatment with antidepressants.

Importantly, the changes in hemorheologic measures that we report in our depressed subjects improved with treatment. Treatment of depression has been reported to lessen the risk of MI associated with depression [32], [33], and MDD patients adequately treated have lower heart disease mortality than inadequately treated depressed patients [33]. The use of antidepressants remains controversial in CVD, especially the use of tricyclic antidepressant medications because they could be associated with an increase risk of CVD [34]. Roose *et al.* [35] have reported in a small controlled trial that paroxetine and nortriptyline are effective treatments for MDD patients with ischemic heart disease. A randomized clinical trial showed that selective serotonin reuptake inhibitor (SSRI) sertraline was safe and effective in treating recurrent depression in patients with an acute MI or unstable angina and without other life-threatening medical conditions [36]. Emerging evidence shows that treatment of MDD patients who experience an acute MI with SSRI antidepressants might decrease mortality or cardiac events [37]. There is evidence that lower adherence to prescribed medications and cardiac prevention measures is present in post-MI patients with depression [38] and that treatment of MDD could improve compliance with CVD treatment. In this study, we examined both a tricyclic and an SSRI antidepressant. Our response rates, which were higher than in some other antidepressant trials, may reflect the fact that placebo responders were eliminated after the first week and that our patients were from a previously untreated, community-based population. A potential concern was the possibility of orthostatic hypotension with desipramine, and subjects in this treatment group did demonstrate more orthostasis by pulse than those treated with fluoxetine (data not shown). However, as described, all of our samples were taken after 5–10 minutes of supine rest. No difference was observed in treatment response or in hemorheologic measures between the two drugs. Figure 1 gives a brief overview on how we could integrate data on acute stress-hemoconcentration and MDD.

Our data based on secoondary analyses support the notion that successful antidepressant treatment ameliorates hemorheologic measures of stress-hemoconcentration, which indicates that factors consistent with increased blood viscosity and the advancement of atherosclerotic plaque in low-pressure sites in the arterial tree may be alleviated following the improvement of MDD symptomatology. Our results suggest that hemorheologic changes in patients suffering from MDD could contribute to an increased risk for CVD. Antidepressant treatment reduces not only the psychological symptoms of depression, but it also may reduce this potentially important depression-associated risk factor for CVD. Hemorheologic measures of stress-hemoconcentration are of low cost and often contained in routine lab assessments, but they have not yet been considered as a relevant biomarker of CVD risk in depression. We suggest here that physicians who assess major depression and provide antidepressant treatment should consider stress hemoconcentration when evaluating the cardiovascular risk factors of depressed patients.

## Supporting Information

Protocol S1 Trial Protocol
(0.06 MB PDF)
