# Supplementary material for: Elevated Stress-Hemoconcentration in Major Depression Is Normalized by Antidepressant Treatment: Secondary Analysis from a Randomized, Double-Blind Clinical Trial and Relevance to Cardiovascular Disease Risk
Source: PLoS One. 2008 Jul 16;3(7):e2350. doi: 10.1371/journal.pone.0002350 (PMC2391294; doi:10.1371/journal.pone.0002350)
Supplement: Protocol S1 — Trial Protocol (0.06 MB PDF) [file pone.0002350.s001.pdf]

University of California, Los Angeles
Office for Protection of Research Subjects
HUMAN SUBJECT PROTECTION COMMITTEE (HSPC)

APPLICATION TO INVOLVE HUMAN SUBJECTS IN RESEARCH

**PROJECT TITLE:** Clinical Pharmacogenetics of Antidepressant Responses in Mexican-Americans				
**PRINCIPAL INVESTIGATOR:**	Name: Julio Licinio	Degree(s): M.D.	University Title: Professor	Campus Phone No.: 310-206-6207
Department: Psychiatry	Campus Mailing Address: 3357A Gonda Bldg		Campus Mail Code: 176122	e-mail Address: licinio@ucla.edu
**CO-INVESTIGATOR or FACULTY SPONSOR:**	Name	Degree(s)	University Title	Campus Phone No.
Department	Campus Mailing Address		Campus Mail Code	e-mail Address
**PRIMARY CONTACT PERSON:**	Name: Fiona O'Kirwan	Campus Phone No.: 310 825 7874	e-mail Address: okirwan@ucla.edu	
**APPLICATION STATUS:**	☐ New ☒ Addendum ☐ Renewal		Previous HSPC number, if applicable: 99-11-049-12	

INVESTIGATOR'S ASSURANCE

I certify that the information provided in this application is complete and correct.

I understand that as Principal Investigator, I have ultimate responsibility for the conduct of the study, the ethical performance of the project, the protection of the rights and welfare of human subjects, and strict adherence to any stipulations imposed by the HSPC.

I agree to comply with all UCLA policies and procedures, as well as with all applicable federal, State, and local laws regarding the protection of human subjects in research, including, but not limited to, the following:

- performing the project by qualified personnel according to the approved protocol,
- implementing no changes in the approved protocol or consent form without prior HSPC approval (except in an emergency, if necessary to safeguard the well-being of human subjects),
- obtaining the legally effective informed consent from human subjects or their legally responsible representative, and using only the currently approved, stamped consent form with human subjects,
- **promptly reporting significant or untoward adverse effects to the HSPC in writing within 5 working days of occurrence.**
- if I will be unavailable to direct this research personally, as when on sabbatical leave or vacation, I will arrange for a co-investigator to assume direct responsibility in my absence. Either this person is named as a co-investigator in this application, or I will advise HSPC by letter, in advance of such arrangements.

____
Principal Investigator Date

FACULTY SPONSOR'S ASSURANCE

By my signature as sponsor on this research application, I certify that the student or guest investigator is knowledgeable about the regulations and policies governing research with human subjects and has sufficient training and experience to conduct this particular study in accord with the approved protocol. In addition,

- I agree to meet with the investigator on a regular basis to monitor study progress.
- Should problems arise during the course of the study, I agree to be available, personally, to supervise the investigator in solving them.
- I assure that the investigator will promptly report significant or untoward adverse effects to the HSPC in writing within 5 working days of occurrence.
- If I will be unavailable, as when on sabbatical leave or vacation, I will arrange for an alternate faculty sponsor to assume responsibility during my absence, and I will advise the HSPC by letter of such arrangements.

____
Faculty Sponsor * (if PI is a student or a fellow) Date

* The faculty sponsor must be a member of the UCLA faculty. The faculty member is considered the responsible party for legal and ethical performance of the project.

SECTION II - FUNDING

THIS SECTION MUST BE COMPLETED

1. Check all of the appropriate boxes for funding sources for this research, include pending funding source(s):

☒Extramural* ☐UCLA Academic Senate ☐Department ☐Gift ☐Other: ___

* P.I. of Contract or Grant: Julio Licinio, M.D.

Funding Source: NIGMS

Contract or Grant No.: 1 U01 GM61394-02

Contract or Grant Title: UCLA Pharmacogenetics and Pharmacogenomics Research Group

2. If using an **IDENTICAL** protocol for more than one extramural funding proposal, list all funding sources below. Attach an additional sheet if more space is needed.

   a. P.I. of Contract or Grant: Dr. Mali Wong

   Funding Source: NIH

   Contract or Grant No.: 1K24RR017365-01

   Contract or Grant Title: Clinical Pharmacogenetics of Antidepressant Responses in Mexican-Americans.

   b. P.I. of Contract or Grant:

   Funding Source:

   Contract or Grant No.:

   Contract or Grant Title:

3. **STATEMENT OF FINANCIAL INTERESTS:** If you are required to submit either a Form 730-U* or a Form 740-U* to the Office of Sponsored Research, please attach a copy of those form(s) with this application. See #9 of the Guidelines for additional information regarding this requirement.

   * Form 730-U, "Principal Investigator's Statement of Economic Interests" for non-governmental funded projects
   * Form 740-U, "Investigator's Statement of Financial Interests" for NSF or PHS funded projects

4. Is this application for the administrative approval for a training grant, a program project, a multiple project grant, or a center grants? ☒Yes ☐No If yes,see Guidelines #14.

If this application is applying for an administrative approval for funding purposes only and does not involve the participation of human subjects, do not complete the rest of this application.

SECTION III - SUMMARY INFORMATION

THIS SECTION MUST BE COMPLETED

The review of research involving human subjects is conducted by either the Medical Human Subject Protection Committee (MHSPC) or the General Campus Human Subject Protection Committee (GCHSPC) depending on the nature of the protocol. The MHSPC is composed of primarily medical specialists, and the GCHSPC has principally socio-behavioral experts and some medical professionals. To aid the OPRS staff in evaluating which HSPC is most likely appropriate for the review of your protocol, please check all appropriate boxes in this section.

1. Will you perform medical procedures as part of this research proposal? ☒Yes ☐No

2. **SUBJECT POPULATION:** (Check all appropriate boxes.)

☐Children (*see Manual Chapters 4,6,8, & 10)*
☐Elderly (*see Manual Chapters 4 & 10)*
☐Fetuses (*see Manual Chapter 8)*
☐Pregnant women (*see Manual Chapter 8)*
☐Terminally ill (*see Manual Chapter 8)*
☐Comatose (*see Manual Chapter 4)*
☐Cancer patients (*see Guidelines #4)*
☒Cognitively or psychologically impaired (*see Manual Chapter 4)*
☐Institutional residents *(see Manual Chapters 4 & 8)*
☐Human in vitro fertilization (*see Manual Chapter 8)*
☐Exclusion of minorities (*see Manual Chapter 8)*
☐Prisoners or parolees (*see manual Chapter8)*
☒Non-English speaking (*see Guidelines #11 & Manual Chapter 8)*
☐UCLA students/staff (*see Guidelines #10 & Manual Chapter 8)*

3. If the research involves any of the following, check the appropriate boxes:

☒Interviews
☒Survey/questionnaire
☐Behavioral observation
☐Deception
☐Waiver of consent
☐Study of existing data *(see Guidelines #12)*
☒Study of human biological specimens *(see Guidelines #12)*
☒Venipuncture (≤450cc)
☒Genetic research
☐HIV/AIDS
☒Clinical studies
☐Investigational drugs *(if checked, complete Section V)*
☐Investigational devices *(if checked, complete Section VI)*
☐Radiation *(see Guidelines #5*)
☐Controlled substances *(see Guidelines #6)*
☐Microorganisms or recombinant DNA *(see Guidelines #7)*
☒Potential development of commercial product from human biological materials *(see Guidelines #8)*
☒PI or Co-PI is the treating physician

4. **LOCATION(S) OF RESEARCH TO BE CONDUCTED AT:**

☒UCLA campus
☐Other locations, specify: ___
☐Santa Monica-UCLA Medical Center

5. **LAY LANGUAGE SUMMARY:** (Please use non-technical language that is understood by nonscientific members to summarize the proposed research project. The information must include: (1) a brief statement of the problem and related theory supporting the intent of the study, and (2) a brief but specific description of the procedure(s) involving the human subjects. Attach an additional page as necessary. However, please do not exceed one single-spaced, type-written page.)

There are 20 antidepressants available on the market, each of which is effective for the treatment of depression leading to acute remission of symptoms in 40-70% of patients. Clinical experience suggests that some of these medications may be particularly helpful to subgroups of patients; there is no clinically accepted method, however, for identifying subjects who should receive particular medications. Pharmacogenomics and pharmacogenetics offer the promise of identification of new genomic targets for antidepressant treatment and may offer the possibility of ascertaining a patient's likelihood of responding to a specific antidepressant based on genetic profiles. This project will intensively study Mexican-American subjects, enrolled in a double-blind treatment trial for major depression, in order to identify pharmacogenetic predictors of treatment response. Mexican-Americans, like any other ethnic group, suffer from depression. However, biological research in depression has never been conducted in this ethnic minority population. Thus, even though Mexican-Americans are the fastest growing minority group in this country, there are no articles in the medical literature on controlled clinical trials in depressed Mexican-Amercians and no studies on the mechanisms of any type of treatment response in this population. It has been hypothesized that associating a particular pattern of genetic polymorphisms with a positive response to a drug may make it possible to define the subset of the population for whom the drug would work best. This hypothesis will be tested in the

following manner. We will conduct a double-blind study of two antidepressants (fluoxetine and desipramine) in a group of 600 Mexican-American subjects (two groups of 300 subjects each). In the course of the study we will conduct detailed and standardized clinical assessments. We will also collect lymphocytes, which will be immortalized and deposited in a DNA bank. As we obtain genetic material and outcome phenotypic data on antidepressant treatment respnses we will determine whether specific genetic factors conribute to pharmacological responses. Because of issues of stratification, it is best to have a population that is as homogenous as possible in genetic studies. The Hispanic population at large is exceedingly diverse, including persons of European, African, Asian, and Native-Amercian background. Genetically, Mexican Amercians are a heterogenous group, with various rates of admixture of European and various Native American genes. Even though the Mexican-American population of Los Angeles is hetergenous, it is less heterogenous than the Hispanic community at large, and certainly less genetically hetergeneous than the overall population of Los Angeles. As it is not possible to obtain over a 5-year period 600 genetically homogeneous, depressed individuals to enter a double-blind depression treatment study in the U.S., we believe that our choice of population decreases genetic stratification as much as possible in a counry as diverse as the United States. To our knowledge there are no other double-blind treatment studies in psychiatry in the Mexican American population, and no pharmacogenetic studies in any area of medicine have been conducted in this population.

SECTION IV – PROTOCOL SUMMARY

INFORMATION REGARDING RENEWAL APPLICATION (1)

1. Renewal Application:
Not Applicable.

PURPOSE OF THE STUDY, THE BACKGROUND AND THE LITERATURE REVIEW (2-3)

2. Purpose of the Study:
This is a study on fluoxetine (Prozac) and desipramine, two different medications that are well known as effective on treating depression. However, every individual responds to a medication differently from another. There is substantial evidence that fluoxetine (Prozac) and desipramine are both effective against depression. However, some people respond to one medication, while others do better with the other. The purpose of this study is to find out whether slight genetic differences (polymorphisms in their DNA sequence) amongst individuals are related to their response to different antidepressant medications

3. Background:
Depression is a common, complex disorder of unknown cause, which affects at least 10% of the U.S. population, resulting in enormous morbidity, considerable mortality, and an economic cost that has been estimated to exceed 50 billion dollars annually. There are 20 FDA approved medications, which are effective in the treatment of depression; however, the likelihood that a specific patient will respond to a specific drug is 40-70%. In fact, although 85-90% of patients eventually respond to antidepressant medication, between 30 and 40% of patients fail to respond to the first antidepressant administered (1). This rate of initial treatment failure is ominous because patients who fail their first antidepressant trial are at increased risk for never getting adequate treatment (2). Elderly patients, as a group, may be at the greatest risk for failure to respond to the initial medication selected, and appear to have greater risk of re-emergent depression once remission is achieved (3), with elder suicide as a tragic consequence in these individuals with untreated depression (4).

The development of an effective predictor of antidepressant treatment response would offer significant clinical advantages. A predictor could be used to identify the most effective medication or class of medications for an individual patient prior to the start of treatment. By increasing the rate of response to the initial antidepressant selected, one could shorten the course of a depressive episode and diminish suffering for a large number of patients. Furthermore, more effective initial treatment of depression could decrease health care costs through decreased utilization of inpatient and outpatient psychiatric services, decreased expenditures on ineffective medication trials, and decreased utilization of general medical services by depressed patients.

A wide variety of possible clinical predictors of antidepressant treatment response have been studied. Factors such as interpersonal relations (5), psychic anxiety (6), past history of conduct disorder (7), co-existing personality disorders (8), focus on somatic concerns (6, 9), delusional features (10), attunement (11), and family history (12) have some value in identifying features that are shared by groups of responders or non-responders, but they have not proven to be reliable pretreatment predictors of response for any given individual patient (13-16). Specifically, none has proven sufficiently useful to be adopted into clinical practice for predicting differential response to medication, that is, which medication is likely to benefit a particular patient.

There is at the present time no marker that is used in clinical practice to identify drugs that would be best for specific patients. Therefore, at this point the choice of antidepressant treatment is based solely on side effect profile. Clinical research studies suggest that symptoms (such as melancholia) (17), pretreatment cerebral metabolism or perfusion (18-21), brain structural features such as white-matter lesions or atrophy

(22-24), or level of quantitative electroencephalographic (QEEG) power in the theta frequency band (25-28) may identify those patients who are most likely to respond to either tricyclic antidepressants (TCAs) or serotoninselective reuptake inhibitors (SSRIs). However, none of those methods offer a clinically accepted tool to determine which specific drug or class of antidepressant drugs should be used in the treatment of an individual patient. Pharmacogenomics and pharmacogenetics offer the promise of identification of new genomic targets for antidepressant treatment and may offer the possibility of ascertaining a patient's likelihood of responding to a specific antidepressant based on her/his genetic profile.

Some molecular targets of potential relevance to the action of currently available antidepressant medications have been identified. These commonly consist of the presynaptic transporter proteins of norepinephrine and/or serotonin, the presynaptic autoreceptors, or the postsynaptic neurotransmitter receptors themselves. In addition, the bioavailability of antidepressant medications to affect central transporters and receptors is significantly affected by the cytochrome P450 (CYP) system in the liver, which is responsible for oxidative metabolism of most antidepressants. It is generally postulated that heterogeneity in these various neurotransmitter transporters or receptors, or in the CYP system for drug metabolism, is related to heterogeneity in medication responsiveness; this construct, however, never has been systematically examined. Moreover, because antidepressant of various classes, acting initially through various monoaminergic pathways exert antidepressant effects after chronic but not acute administration, it has been hypothesized that novel downstream genomic targets for antidepressant action may exist in the central nervous system.

There is a vast literature supporting that depression is a risk factor for cardiovascular disease. We have a new NIH grant specific aimed at studying this important topic (grant #.....). A review by Glassman, AH and Shapiro, PA (Am J Psychiatry, 1998; 155:4-11) summarizes well this area. The link between depression and premature death has been suspected by clinicians and folk wisdom for a long time. This scientific question started to be addressed in 1970s; investigators compared mortality among patients treated for major depression and the general population. Ninety percent of the studies found an increased mortality from cardiovascular disease among depressed patients, these association persisted even after controlling for the effects of antidepressant treatment and for the effects of cigarette smoking. Not only did a relation between depression and mortality persist, but a relation between depression and the development of ischemic disease was revealed. This finding was replicated by several studies, community surveys have observed an increased risk of ischemic heart disease among depressed persons. Another research strategy that has been used is to study subjects who have preexisting cardiovascular disease. Here, too, depression has consistently been associated with a worse outcome. In one carefully-designed study, patients with depression in the period immediately after a myocardial infarction were 3.5 times more likely to die than nondepressed patients.

Genetic controls

The purpose of this study is to find out whether slight genetic differences (polymorphisms in their DNA sequence) that are associated with response to different antidepressant medications are also associated with a diagnosis of depression. We want to determine if there is an overlap in genetic markers for depression and genetic markers for antidepressant treatment response.

Cardiovascular controls

In order to determine whether there is a diagnosis effect we propose to have a control group in whom depression has been excluded and who match the patients in this study by demographic and anthropometric variables. It is a scientific necessity to have such a control group in order to determine whether the proposed outcome measures are affected by the diagnosis of depression. Changes in hormones, lipids, and clotting factors may increase a person’s risk for cardiovascular disease. In order to determine whether these substances are affected by depression blood samples can be compared between depressed and non-depressed individuals. Lipid and coagulation factors in age sex and BMI matched healthy controls will be compared to depressed patients.

CHARACTERISTICS OF THE SUBJECT POPULATION (4-6)

4. Number of Subjects:

Depressed subjects
600 subjects, ages 18-70 years, with phenotypically-defined major depressive disorder, according to DSM IV criteria (30), will be enrolled in the study.

Genetic Controls
200 DNA control subjects, ages 18-70 years, with or without major depressive disorder.

Cardiovascular Controls
80 Lipid and Coagulation control subjects, ages 18-70 years, non-depressed (as defined by DSM IV criteria(30)) will be enrolled in the study

5. Inclusion/Exclusion Criteria:

Depressed subjects

Inclusion Criteria

1. Adult Mexican-American men and women between 18 and 70 years of age with major depression are eligible to participate in this study. For the purpose of the study, Mexican-Americans are individuals who have at least 3 out of 4 grandparents born in Mexico.

Exclusion Criteria:

Subjects will not be able to participate in this study if they meet one or more of the following exclusion criteria:

1. If they are on medications with significant activity on the brain, like benzodiazepines (e.g. Valium), or they are in active treatment with an antidepressant medication that cannot be discontinued.

2. If they have any other serious medical illness or chronic psychiatric disorder.

3. If they are pregnant, trying to become pregnant, breast-feeding an infant or sexually active and not using effective contraception.

4. If illegal or illicit drugs are detected in their blood or urine and/or they are current illicit drug users.

5. If they have any active medical illness that may be related to the ongoing depression, like untreated hypothyroidism or stroke within the past 6 months.

6. If they have been receiving electroconvulsive therapy (ECT) in the past 6 months. 6. Vulnerable Subjects

Genetic Controls

Inclusion Criteria

1. Adult Mexican-American men and women between 18-70 years of age are eligible to participate in this study. For the purpose of the study, Mexican-Americans are individuals who have at least 3 out of 4 grandparents born in Mexico.

Exclusion Criteria

None.

Cardiovascular Controls

Inclusion Criteria

1. Adult Mexican-American men and women between 18-70 years of age, healthy, non-depressed individuals are eligible to participate in this study. For the purpose of the study, Mexican-Americans are individuals who have at least 3 out of 4 grandparents born in Mexico.

Exclusion Criteria

None.

6. Vulnerable Subjects:

Depressed Subjects

Mexican-American patients (ages 18-70) with major depressive disorder, according to DSM IV criteria (30), will be recruited for this study. A large portion of subjects are Spanish speaking only, however all members of clinic staff are highly trained and bilingual to ensure that Spanish speaking subjects receive the same standard of care as a non-vulnerable population. Subjects are asked which language they wish to converse in and consent forms are provided in Spanish and English formats and are thoroughly explained.

Even though Mexican Americans are the fastest growing minority group in this country, there are in the medical literature no articles on controlled clinical trials in depressed Mexican Americans and no studies on the mechanisms of any type of treatment response in this population. Our population will be by no means homogenous, but it will certainly be less genetically heterogeneous than the overall population of the Los Angeles area. As there are no genetically isolated populations in the United States that could provide over five years 600 subjects for a double-blind study of the pharmacogenetics of antidepressant treatment. We believe that our focus will decrease the genetic heterogeneity that would be encountered by mixing together in this study all ethnic groups that live in the Los Angeles metropolitan region.

Genetic Controls

Mexican-American subjects with or without major depressive disorder. A large portion of subjects are Spanish speaking only, however all members of clinic staff are highly trained and bilingual to ensure that Spanish speaking subjects receive the same standard of care as a non-vulnerable population. Subjects are asked which language they wish to converse in and consent forms are provided in Spanish and English formats and are thoroughly explained.

Cardiovascular Controls

Adult Mexican-American men and women between 18-70 years of age, healthy, non-depressed individuals are eligible to participate in this study. A large portion of subjects are Spanish speaking only, however all members of clinic staff are highly trained and bilingual to ensure that Spanish speaking subjects receive the same standard of care as a non-vulnerable population. Subjects are asked which language they wish to converse in and consent forms are provided in Spanish and English formats and are thoroughly explained.

SUBJECT IDENTIFICATION AND RECRUIMENT (7)

7. Method of Subject Identification and Recruitment:

Depressed Subjects

Subjects will be recruited through referrals from UCLA Medical Center and primary care physicians. We will distribute fliers about depression and about the study at UCLA clinics where large numbers of depressed patients are treated. We will also distribute flyers in the community. We will use newspaper advertising in local and Spanish language newspapers. When potential subjects call the UCLA Clinic non qualified individuals will be screened out using a "Consent Script To Screen For Research". An appointment to visit the clinic for a psychiatric screening (using the SCIDS) and physical exam will be made for potential subjects who pass the phone screening.

Genetic Controls

Control subjects will be recruited through referrals from UCLA Medical Center and primary care physicians. We will distribute fliers about participating as a controls subject at UCLA Medical Center and in the community.

Cardiovascular Controls

Cardiovascular control subjects will be recruited through referrals from UCLA Medical Center and primary care physicians. We will distribute fliers about participating as a control subject. We will also use newspaper advertising in local and Spanish language newspapers. When potential subjects call the UCLA Clinic non qualified individuals will be screened out using a "Consent Script To Screen For Research". An appointment to visit the clinic for a psychiatric screening (using the SCIDS) and physical exam will be made for potential subjects who pass the phone screening.

METHODS AND PROCEDURES (8-11)

8. Methods and Procedures Applied to Human Subjects:

Depressed Subjects

This is a prospective treatment study with single- and double-masking, with random assignment of treatment, and with outcomes assessed with clinical and functional measures. All subjects will undergo a comprehensive intake assessment, and then follow-up assessments and treatment performed under the protocol. All subjects will undergo a one-week single-masked placebo lead-in, as it is a standard procedure in pharmacological studies of major depression. All subjects still meeting entry criteria at the end of the placebo lead-in then will be assigned to active double-masked treatment with either fluoxetine or desipramine. Treatment will be continued for eight weeks on an outpatient basis. Dosage will be increased at weeks 4 and 6 for non-responders. This is designated to parallel the clinical practice of dose adjustment in patients who are not showing adequate clinical responses.

The presence of specific clinical symptoms will be determined with the Structured Clinical Interview for Diagnosis (SCID) instrument (29). The SCID instrument operationalizes the DSM IV diagnostic criteria for major Axis I disorders, and the presence of a current depressive episode will be determined with this instrument. For some diagnoses not covered by the SCID, i.e. dementing illnesses, the DSM-IV criteria (30) will be provided in checklist form to the interview to assist in detecting any excluded diagnoses.

At entry into the study, subjects will complete the Beck Depression Inventory (BDI; Beck, 1961) a self-report of the presence and severity of depressive symptoms. Subjects will be rated on the Hamilton Depression (Ham-D) (31) and Hamilton Anxiety Rating Scales (HAM-A) (32) and the Global

Assessment Scale (GAS) (33). The Ham-A scale provides clinician-rated data on the presence and severity of anxiety symptoms. The GAS provides an overall assessment of a subject's level of functional impairment. Intake into the study requires Ham-D values to be above threshold at initial presentation and after the one week, single-masked placebo lead-in phase.

Optional Psychiatric Screening visit:
Some subjects may complete the psychiatric component of the screening in the community or their home before they will be asked to travel to UCLA for further medical screening. The interviewer will travel to a community site or the patient’s home to perform the evaluation.

Other evaluations will include a history taking session, and a physical and neurological examination, checking of vital signs (blood pressure, heart rate, body temperature and breathing rate) along with laboratory studies of blood and urine of substances that may be affected by depression such as hormones lipids, and clotting factors. Those tests will be done at UCLA labs (Clinical Pathology, Dr. Licinio’s lab, and Dr. Willa Hseuh’s lab), Dr. Victor Marder’s lab at Orthopedic Hospital, and at collaborating centers such as Childrens Hospital Oakland Research Institute(Dr. Ronald Krauss’s lab). Blood and urine, including tests for the presence of illegal or illicit drugs including alcohol. For that purpose about 10 tablespoon (or 150 cc) of blood will be drawn from a vein in the patient's arm. Some of this blood will be used for genetic analysis. An electrocardiogram (ECG) will be taken to determine heart rate, and women will be tested for pregnancy if needed. The subject will be asked to provide information about past and present illnesses and list any medication he/she is currently taking, including herbal and over-the-counter drugs. The results of these studies will be discussed with the subject. In case he/she is currently on any medication for mood, sleep or anxiety disorders, investigators will contact his/her primary care physician or the doctor who is prescribing that medicine, before stopping the medication. Once a subject is enrolled in the study, he/she must contact the study investigator or his/her assistant before taking any other medication besides those that are the object of the study. Subjects enrolled in the study will not be able to start or continue any psychotherapy while they are participating in the study. Laboratory studies of blood and urine will be repeated further in the study, according to the flow chart below. The maximum total amount of blood to be drawn throughout the study will not exceed 24 tablespoons (or 350 cc).

Subjects will undergo frequent ratings, with follow-up visits scheduled at 1 week after intake (i.e. at the end of placebo lead-in) and weekly thereafter. Clinical status will be assessed at all follow-up visits with clinical interview and rating with the Ham-D (31), BDI (34), and Ham-A (32), and several functional status and quality of life instruments.

Several measures of social functioning have been shown to be sensitive to social dysfunction in depression. The main efficacy measure is the derived 17-item score from the 25-item Hamilton Rating Scale for Depression (HAM-D-25). Other efficacy measures in this study include the Medical Outcomes Study Short Form 36 (SF-36) (35) and the Center for Epidemiologic Studies Depression Scale (CES-D) (36). These measures will allow a thorough characterization of subjects. These efficacy measures will be administered according to the study flow-chart (attached to end of HS1 form).

The subjects will be assigned randomly to receive either fluoxetine or desipramine for an 8 week, double-masked phase. Subjects randomized to fluoxetine will initially receive a 10 mg/day dose increasing to 20mg/day at week 2. At four weeks, an assessment divides subjects into responders (who continue to receive 20 mg (solid line), and partial or non-responders (who escalate to 40 mg max(i.e., less than a 25% decrease in Ham-D score) (16). Subjects will be followed by a clinician on weekly appointments (see flow chart). After 8 weeks on fluoxetine, subjects will be discharged from this study to other acute treatment studies (in the case of non-responders or partial responders), or continuation treatment studies (in the case of responders) if such studies are available at UCLA clinical center. Parallel procedures will be followed for desipramine 50/100/200 mg. Subjects and clinicians will be aware of the dose escalation, but will not know which agent is being employed.

The dose escalation will be carried out according to the following protocol. The dose will be increased at week 4 by 10mg fluoxetine/50mg desipramine for patients who show less than a 25% decrease in Hamilton Depression Score. If at week 6 the Hamilton Depression Score is greater than 12 the dose will be increased by 10mg fluoxetine/50mg desipramine resulting in a maximum dose of 40mg fluoxetine/ 200mg desipramine.

At the end of the 8-week double-masked phase, each subject will be assessed to determine degree of response. Subjects who are "responders" are those who exhibit a final Ham-D score of less than or equal to 10. For research purpose only, subjects who fail to exhibit a complete response to active treatment with one agent will be divided into two groups to characterize the nature of their response to treatment: "non-responder" and "partial responder" groups. The partial responder subjects are those who complete the double-masked phase with a Ham-D score of greater than 10 but who showed a decrease in Ham-D score of at least 25% (e.g. falling from 24 to 17); any lesser degree of change will place a subject in the complete non-responder group.

Subjects will be referred out of the study at the end of the 8 week controlled trial. Primary psychiatric care for these individuals after the follow-on phase will be provided by a physician of the subject's choice; this physician will prescribe and monitor medications and make dosing and other adjustments on the basis of his or her clinical judgment. Subjects who did not respond or were partial responders may be referred either to standard care or to other research studies.

Subjects may be removed at several places in the protocols. Subjects who exhibit a placebo response during the lead-in period, which either is significant or would diminish the severity of illness below entry criteria will be removed and replaced; they will be referred for open label treatment in the clinic. Placebo response is defined as a drop in Ham-D to 17 or below and/or a decrease of 25% or more on the Ham-D score. Subjects who report intolerable side effects have the prerogative to leave the protocol and will be replaced to maintain the experimental design. Subjects who develop suicidal intent or plan will be removed from the protocol, informed of the treatment assigned, and referred for open-label treatment, as will subjects who develop manic symptoms and those who develop a sustained increase in blood pressure (a potential side effect of desipramine). At any point, subjects may elect to leave the protocol, and under such circumstances, they will be replaced with another recruited subject.

In summary, agents used in this protocol will be:

• fluoxetine (Prozac), a serotonin reuptake inhibitor.

• desipramine, a noradrenergic reuptake inhibitor.

• placebo (for one week, as a wash-in, only). There will be no placebo treatment per se in this study. Because extensive double-blind placebo studies have already been done with fluoxetine and with desipramine it would not be ethical to withhold active treatment for 8 weeks in symptomatic depressed patients.

Matching fluoxetine, desipramine, and placebo study materials will be compounded by the UCLA Pharmacy. Fluoxetine will be administered at 20 mg per day and desipramine at 100 mg per day during the initial four weeks of active treatment. For subjects not showing significant response by 4 weeks (as described above), the dose will be further escalated to 40 mg a day of fluoxetine or 200 mg of desipramine.

Optional Treatment Course
If the subject experiences severe side effects within the first 3 weeks of the study they will be given the following treatment options.
1. Discontinue the study and the medication.

2. Discontinue the current medication and start the other medication in this study. If they choose this option the course of treatment will begin again at week 1 and they will continue the study for another 8 weeks.

Media consent process
It has never been the policy at UCLA to allow a representative of the media to contact a patient directly. Therefore, we shall continue to provide a consenting subject with the contact information for the media and to allow the subject to initiate the contact if he or she so desires.

When a representative of the media requests to interview a subject, we will:
- Inform the subject by phone of the media's interest in conducting interviews;
- Provide the subject with contact information for Israel Alvarado, M.D., (310) 206 6051, and clinic staff at (310) 206 3653 who can further discuss the media request(s), explain the interview process, and answer any questions that the subject may have.
- Provide the subject with a copy of the media interview consent form to review, allowing them sufficient time to review the form;
- Ensure the subject that his or her decision to participate in the media interview will in no way affect their future relationship with UCLA;
- Once written consent has been obtained from interested subjects, will provide them with the appropriate contact information for the Univision representative or if they prefer we will contact the representative and set up the interview at a time that is convenient for them.

The following script that will be used to inform subjects about media interest in the research:

- The television station Univision has expressed an interest in the depression study in which you participated at UCLA. They would like to interview some subjects that participated in this study in order to inform the public about the research.
- Would you be interested in speaking to someone from Univision about your participation in this study? I would like to assure you that your decision one way or another will have absolutely no impact on your future participation in any study at UCLA, and will in no way affect your relationship with the study staff or with me.
- If you do decide you would like to be interviewed, the interview will be at our clinic here at UCLA that you attended during your participation in the study. We will do everything we can to make the interview at a time that is convenient for you.
- If you decide you would like to participate in the interview, you will be given the name and telephone number of the interviewer. You may either make the contact yourself, or Israel Alvarado, M.D., or clinic staff will arrange initial contact for you. Rest assured no media representative would have access to your telephone number unless you choose to give your number to the interviewer.
- Take some time to think about this. If you decide you would like to participate in an interview, or if you have questions that you would like to have answered before you make your decision, please call Israel Alvarado, M.D., (310) 206 6051, or clinic staff at (310) 206 3653.

Please let me know if you have any questions. Thank you.

<u>Genetic Controls</u>

Approximately 3-4 tablespoon (or 50 cc) of blood will be drawn from a vein in the patient's arm.

<u>Cardiovascular Controls</u>
A complete medical and psychiatric evaluation will be performed. The psychiatric assessment involves determining the presence of specific clinical symptoms with the Structured Clinical Interview for Diagnosis (SCID) instrument (29). The SCID instrument operationalizes the DSMIV diagnostic criteria for major Axis I disorders, and determines the presence of a current depressive episode.

As part of the medical evaluation the control subjects will be asked to provide information about past and present illnesses and list any medications they are taking including herbal medications and over the counter medications. The medical evaluations will include a physical and neurological examination and checking of vital signs. An electrocardiogram will be taken to determine heart rate.

We will draw about ten tablespoons (or 150cc) of blood from a vein in the subjects arm. This blood will be used to analyze substances such as hormones, lipids and clotting factors. Urine will be tested for the presence of illegal drugs including alcohol.

The complete medical and psychiatric evaluation, and obtaining blood sample will take about 2.5 -3 hours.

9. For Research Involving Survey, Questionnaires, etc:

<u>Depressed Subjects</u>

The psychiatric assessment involves determining the presence of specific clinical symptoms with the Structured Clinical Interview for Diagnosis (SCID) instrument (29). The SCID instrument operationalizes the DSMIV diagnostic criteria for major Axis I disorders, and determines the presence of a current depressive episode.

<u>Genetic Controls</u>

No instruments are involved.

<u>Cardiovascular Controls</u>

The psychiatric assessment involves determining the presence of specific clinical symptoms with the Structured Clinical Interview for Diagnosis (SCID) instrument (29). The SCID instrument operationalizes the DSMIV diagnostic criteria for major Axis I disorders, and determines the presence of a current depressive episode.

10. FDA Approval:

Fluoxetine and desipramine are FDA approved.

11. Data Collection, Storage and Confidentiality:

<u>Depressed Subjects / Genetic Controls / Cardiovascular Controls</u>

The only people who will know that a patient is a research subject are members of the research team and, if appropriate his/her physicians and nurses. No information about the patient, or provided by him/her during the research, will be disclosed to others without his/her written permission, except if it is necessary to protect his/her rights or welfare (for example, if he/she is injured and need emergency care), or if it is required by law.

When the results of the research are published or discussed in conferences, no information will be included that would reveal the patient's identity. If photographs, videos, or audiotape recordings are used for educational purposes, his/her identity will be protected or disguised. A11 data will be kept in locked files, and patients will be identified by codes when the data gathered in this procedure is presented or published.

Authorized representatives of the Food and Drug Administration (FDA) or a funding agency, such as the National Institutes of Health may need to review records of individual subjects. As a result, they may see patients' names; but they are bound by rules of confidentiality not to reveal their identity to others.

By signing the consent form, the subject allows the study investigators to determine what to do with any surplus tissue (blood) removed from him/her during the study; this includes DNA, which is everyone's

genetic material. Each person has his or her own unique DNA sequence, which is like a fingerprint. The purpose of this research is to examine whether DNA sequence is related to treatment response. Patients' DNA and treatment responses will be deposited in a "DNA Bank" at UCLA for distribution to other researchers and also at a national database created by the National Institute of General Medical Sciences (NIGMS), National Institutes of Health. Patients' DNA sequences and treatment responses will be shared with others. When we share any subject's information with other parties his/her name and personal identifying information will be kept confidential; however, it is possible that DNA sequence, like a fingerprint, might one day be traced back to the subject's identity. Because each person has his/her own and unique genetic sequence, there is the possibility that a unique genetic sequence might be used to track a subject's name or other health and familiar (genealogical) relations.

There are risks that a subject is exposed to when his/her health and genetic information are stored in a data bank. Health records have been used against patients and their families. For example, insurance companies may deny a patient insurance or employers may not hire someone with a certain illness (such as AIDS or cancer). The results of genetic research may not apply only to the individual, but to his/her family members too. For diseases caused by genetic changes, the information in one person's health record could be used against family members.

By signing the consent form, the subject will give his/her permission to the investigators to share all information collected, including his/her genetic sequence and other genetic information with other researchers. The subject's information could be used in future research projects that are entirely unrelated to depression or medications.

All blood analysis results and genetic information will be kept in locked cabinet files and restricted-access computers. They will be kept separate from records with the patient's name and other personal information. There is a slight possibility that a patient's name might be related to his/her genetic and/or other personal record collected during the study. The investigators will use all the precautions permitted by law, policy and technology to keep everyone's personal identity and name secret. However, complete confidentiality might not be reached.

The investigators related to this study will inform every subject about any economic interest they have in this research. Investigators are not required to store his/her samples indefinitely.

Cells obtained from participants' bodies may be used to establish cell lines which may be shared in the future with other researchers and which may be of commercial value. A cell line is one that will grow indefinitely in the laboratory. Cell lines may be useful because of the characteristics of the cells and/or the products they may produce. All tissue and/or fluid samples are important for this research study. All samples and information will be in the public domain, meaning that any person, company, or government will not own it. If a commercial product is developed from your information or samples, the commercial product will be owned by the manufacturer or inventor of the product.

State laws require mandatory reporting on such issues as homicidal or suicidal intents and child abuse. In case these circumstances occur, pertinent authorities may request the subject's records without his/her previous consent. Information regarding suicidal attempts, homicidal ideation, and child abuse may be shared, if necessary, with other physicians outside the study or the appropriate authorities.

RISK / BENEFIT ASSESSMENT (12-17)

12. Potential Risk and Discomforts:

Depressed Subjects

The procedures listed above may result in discomforts or inconveniences. The known risks, inconveniences or side effects involved in this study are the following:

1. The patient will be taking an ineffective medicine (placebo) for the first week during the study.

2. The study medication the patient is assigned to may not work and his/her depression may worsen. Consequences could be hospitalization and problems to his/her family, job or finances.

3. List of possible side effects from fluoxetine (Prozac) and desipramine is long, but they are not usually important. They include nausea, diarrhea, low appetite, headache, restlessness, poor or too much sleep, itching, rash, dry mouth, constipation, dizziness, tiredness, and difficulties achieving orgasm. Desipramine might also raise blood pressure.

4. There is always the possibility that unknown adverse or side effects might occur during the study. The study investigator will watch carefully for unexpected reactions.

5. The side effects of drawing blood may be some pain, bruise or bleeding at the time the needle is inserted. Serious adverse effects are blood cot or infection, but they are very rare. Some people may faint during the procedure.

Genetic Controls
The side effects of drawing blood may be some pain, bruise or bleeding at the time the needle is inserted. Serious adverse effects are blood cot or infection, but they are very rare. Some people may faint during the procedure.

Cardiovascular Controls

The side effects of drawing blood may be some pain, bruise or bleeding at the time the needle is inserted. Serious adverse effects are blood cot or infection, but they are very rare. Some people may faint during the procedure.
There is a risk of emotional discomfort when answering personal questions during the psychiatric evaluation.

13. Risk Classification:
Depressed Subjects
The present study cannot be classified as minimal risk.
Genetic Controls
Minimal risk
Cardiovascular Controls
Minimal risk

14. Minimizing Risks:

Depressed Subjects
All potential risks and discomforts will be minimized by weekly monitoring by participating investigators. Subjects are questioned thoroughly about side effects at each visit to minimize risk due to side effects. Research subjects will be immediately withdrawn from the study upon evidence of any adverse event.

Optional Treatment Course
If the subject experiences severe side effects within the first 3 weeks of the study they will be given the following treatment options.

1. Discontinue the study and the medication.

2. Discontinue the current medication and start the other medication in this study. If they choose this option the course of treatment will begin again at week 1 and they will continue the study for another 8 weeks.

Staff members are trained in conducting the psychiatric interviews to minimize any emotional discomfort.

Genetic Controls
Only trained staff members will draw the blood and apply pressure to minimize bleeding

Cardiovascular Controls
Only trained staff members will draw the blood and apply pressure to minimize bleeding. Staff members are trained in conducting the psychiatric interviews to minimize any emotional discomfort.

15. Potential Benefits:

Depressed Subjects
ANTICIPATED BENEFITS TO SUBJECTS
We do not expect this study to be of direct benefit to the participants. It is possible that their depression may improve during the study. However, as they will receive treatment for about 10 weeks, those benefits may not last and they will need further treatment not included in this study.

ANTICIPATED BENEFITS TO SOCIETY
The results we obtain will give us information on why medications work differently in different individuals. That could bring to more effective treatment of depression and other illnesses with fewer adverse effects.

Genetic Controls
ANTICIPATED BENEFITS TO SUBJECTS
We do not expect this study to be of direct benefit to the participants.

ANTICIPATED BENEFITS TO SOCIETY
The results we obtain will give us information on why medications work differently in different individuals. That could bring to more effective treatment of depression and other illnesses with fewer adverse effects.

Cardiovascular Controls

ANTICIPATED BENEFITS TO SUBJECTS
We do not expect this study to be of direct benefit to the participants.

ANTICIPATED BENEFITS TO SOCIETY
The results we obtain will give us information on why medications work differently in different individuals. That could bring to more effective treatment of depression and other illnesses with fewer adverse effects.

16. Therapeutic Alternatives:
Depressed Subjects
Participation in this study will be entirely voluntary. Subjects can choose not to participate in the study. Patients will be referred to UCLA Clinical Center for proper treatment. An alternative to treatment with medication is psychological counseling.

Genetic Controls
Participation in this study will be entirely voluntary. Subjects can choose not to participate in the study.

Cardiovascular Controls
Participation in this study will be entirely voluntary. Subjects can choose not to participate in the study.

17. Risk / Benefit Ratio:

Depressed Subjects
There is no unreasonable risk in this study. No experimental or FDA non-approved medicines will be involved. Patients may improve their depressive symptoms as a result of treatment, and will be at the same risk of other people not involved in the study and taking the same medications. The risk of loss of confidentiality is balanced by the opportunity to have a well conduct trial of pharmacologically effective antidepressant treatment.

Genetic Controls
There is no unreasonable risk in this study.

Cardiovascular Controls
There is no unreasonable risk in this study.

FINANCIAL CONSIDERATION (18-20)

18. Payment for Participation:

Depressed Subjects
Medication (fluoxetine or desipramine) will be provided to every subject free of charge during the study. A $20.00 per visit reimbursement for transportation costs and a $50 bonus for the final visit will be provided.

Genetic Controls
Subjects will be reimbursed $50 for transportation costs.

Cardiovascular Controls
Subjects will be reimbursed $50 for transportation costs.

19. Financial Obligations of the Subjects:
Neither the subject nor his or her insurance company will be billed for participation in this research. Other medical care, which is not scheduled for this study, may be charged to the patient or his/her insurance company

20. Emergency Care and Compensation for Research-Related Injury:
If anyone is injured as a direct result of research procedures not done primarily for one's own benefit, he or she will receive treatment at no cost
.

INFORMED CONSENT (21-26)

21. Capacity of Consent:
All subjects enrolled in the study will have the capacity to consent. We will use the capacity to informed consent tool.

22. Personnel Inviting Participants:
Individuals authorized to solicit consent and sign the consent form confirming that the prospective subject was provided the necessary information and that any question asked was answered are enlisted below:

Name	Address	Daytime Phone	Night/EmergencyPhone
Adrian Llerena, M.D.	UCLA 1554 Gonda Bldg	310-206-6628	310-825-0511
Tuncay Delibasi, M.D.	UCLA C8-255 NPI	310-794-9981	310-825-0511
Anil Sharma, M.D.	UCLA C8-243 NPI	310-206-0051	310-825-0511
Julio Licinio, M.D.	UCLA 3357A Gonda Bldg	310-206-6207	310-825-0511
Ma-Li Wong, M.D.	UCLA 3357B Gonda Bldg	310-206-6123	310-825-0511

23. Process of Consent:
Depressed Subjects
Process of consent will take place at UCLA Clinical Center during first outpatient visit.
Genetic Controls
Process of consent will take place at UCLA Clinical Center
Cardiovascular Controls
Process of consent will take place at UCLA Clinical Center

24. Comprehension of the Information Provided:
Potential participating subjects' comprehension about the research will be assessed by questioning them on the various items of the consent form. Non-English speaking subjects will be interviewed by an investigator fluent both in English and Spanish, and will receive a Spanish translation of the consent form. There are validated assessment tools in Spanish available.

25. Information Withheld From Subjects:
No information about the research purpose and design will be withheld.

26. Consent / Assent Forms:

Depressed Subjects
Adult consent form titled "Clinical Pharmacogenetics of Antidepressant Responses in Mexican-Americans Lay Title: A study of two Medications for Depression in Mexican-Americans" will be used in English and Spanish formats

Genetic Controls
Adult consent form titled "Clinical Pharmacogenetics of Antidepressant Responses in Mexican-Americans Lay Title: Studies of Genetic Variations in Mexican –Americans control subject consent form" will be used in English and Spanish formats

Cardiovascular Controls

Adult consent form titled "Cardiovascular Disease Biological Markers in Depressed Persons of Mexican Origin Control Subject Consent Form" will be used in English and Spanish formats.

REFERENCES:

1. Baldessarini RJ. Current status of antidepressants: clinical pharmacology and therapy. J Clin Psychiatry 1989;50: 117-26.

2. Katzelnick D, Kobak, K, Greist, JK, Jefferson, JW, Henk, HJ. Predictors of adequate dose and duration of antidepressant medication for depression in a HMO. In: New Clinical Drug Evaluation Unit (NCDEU), 37th Meeting; 1997; Boca Raton, FL, May 2730; 1997. p. Poster 86.

3. Hinrichsen GA. Recovery and relapse from major depressive disorder in the elderly. Am JPsychiatry 1992;149:1575-9.

4. Conwell Y. Suicide in elderly patients. In: Schneider L, Reynolds, CF III, Lebowitz, BD, Friedhoff, AJ, editor. Diagnosis and Treatment of Depression in Late Life: Results of the NIH Consensus Development Conference. Washington, DC: American Psychiatric Press; 1994.

5. Blatt SJ, Quinlan DM, Zuroff DC, Pilkonis PA. Interpersonal factors in brief treatment of depression: further analyses of the National Institute of Mental Health Treatment of Depression Collaborative Research Program. J Consult Clin Psychol 1996;64:162-71.

6. Burns RA, Lock T, Edwards DR, Katona CL, Harrison DA, Robertson MM, et al. Predictors of response to amine-specific antidepressants. J Affect Disord 1995;35:97-106.

7. Rowe JB, Sullivan PF, Mulder RT, Joyce PR. The effect of a history of conduct disorder in adult major depression. J Affect Disord 1996;37:51-63.

8. Fava M, Bouffides E, Pava JA, McCarthy MK, Steingard RJ, Rosenbaum JF. Personality disorder comorbidity with major depression and response to fluoxetine treatment. Psychother Psychosom 1994;62: 160-7.

9. Hoencamp E, Haffmans PM, Duivenvoorden H, Knegtering H, Dijken WA. Predictors of (non-) response in depressed outpatients treated with a three-phase sequential medication strategy. J Affect Disord 1994;31:235-46.

10. Bielski RJ, Friedel RO. Prediction of tricyclic antidepressant response: a critical review. Arch Gen Psychiatry 1976;33:1479-89.

11. Geerts E, Bouhuys N, Van den Hoofdakker RH. Nonverbal attunement between depressed patients and an interviewer predicts subsequent improvement. J Affect Disord 1996;40: 15-21.

12. Hinrichsen GA, Hernandez NA. Factors associated with recovery from and relapse into major depressive disorder in the elderly. Am J Psychiatry 1993;150:1820-5.

13. Mulder RT, Joyce PR, Sullivan PF, Oakley-Browne MA. Intimate bonds in depression. J AffectDisord 1996;40:175-8.

14. Small GW, Hamilton SH, Bystritsky A, Meyers BS, Nemeroff CB. Clinical response predictors in a double-blind, placebo-controlled trial of fluoxetine for geriatric major depression. Fluoxetine Collaborative Study Group. Int Psychogeriatr 1995;7:41-53.

15. Cohn JB, Wilcox C. A comparison of fluoxetine, imipramine, and placebo in patients with major depressive disorder. J Clin Psychiatry 1985;46:26-31.

16. Nierenberg AA, McLean NE, Alpert JE, Worthington JJ, Rosenbaum JF, Fava M. Early nonresponse to fluoxetine as a predictor of poor 8-week outcome. Am J Psychiatry 1995;152: 1500-3.

17. Nobler MS, Roose SP. Differential response to antidepressants in melancholic and severe depression. Psychiatric Ann 1998;28:84-8.

18. Nobler M, Sackeim, HA, Louie, J, Prohovnik, I, Roose, SP, Van Heertum, R. Clinical response to antidepressants is associated with reduced frontal CBF in late-life depression. In: The One-Hundred and Fifty-First Annual Meeting of the American Psychiatric Association; 1998; Toronto, Ontario, Canada, May 30-June 4; 1998. p. abstract No. NR372.

19. Kennedy S, Evans, KR, Houle, S, Vaccarino, FJ. PET analysis of antidepressant effectsof paroxetine. In: The One-Hundred and Fifty-First Annual Meeting of the American Psychiatric Association; 1998; Toronto, Ontario, Canada, May 30-June 4; 1998. p. abstract No. 47.

20. Sackeim HA, Prohovnik I, Moeller JR, Brown RP, Apter S, Prudic J, et al. Regional cerebral blood flow in mood disorders. I. Comparison of major depressives and normal controls at rest. Arch Gen Psychiatry 1990;47:60-70.

21. Sackeim HaP, I. Studies of brain imaging in mood disorders. In: Mann JaK, DJ, editor. The Biology of Depressive Disorders. Part A: A System Perspective. New York, NY: Plenum; 1993. p. 205-58.

22. Figiel GS, Krishnan KR, Breitner JC, Nemeroff CB. Radiologic correlates of antidepressant-induced delirium: the possible significance of basal-ganglia lesions. J Neuropsychiatry Clin Neurosci 1989;1:188-90.

23. Figiel GS, Krishnan KR, Doraiswamy PM. Subcortical structural changes in ECT- induced delirium. J Geriatr Psychiatry Neurol 1990;3: 172-6.

24. Hickie I, Scott E, Mitchell P, Wilhelm K, Austin MP, Bennett B. Subcortical hyperintensities on magnetic resonance imaging: clinical correlates and prognostic significance in patients with severe depression. Biol Psychiatry 1995;37:151-60.

25. Ulrich G, Haug HJ, Stieglitz RD, Fahndrich E. Are there distinct biochemical subtypes of depression? EEG characteristics of clinically deEmed on-drug responders and non- responders. J Affect Disord 1988;15:181-5.

26. Ulrich G, Haug HJ, Stieglitz RD, Fahndrich E. EEG characteristics of clinically defined on-drug-responders and non- responders--a comparison clomipramine vs. maprotiline. Pharmacopsychiatry 1988 ;21:367-8.12

27. Ulrich G, Haug HJ, Fahndrich E. Acute vs. chronic EEG effects in maprotiline- and in clomipramine- treated depressive inpatients and the prediction of therapeutic outcome. J Affect Disord 1994;32:213-7.

28. Knott VJ, Telner JI, Lapierre YD, Browne M, Horn ER. Quantitative EEG in the prediction of antidepressant response to imipramine. J Affect Disord 1996;39: 175-84.

29. First M, Spitzer, RL, Gibbom, M, Williams, JB. Structured Clinical Interview for Axis I DSM-IV Disorders-Patient Edition (SCID-I/P, version 2). New York, NY: Biometrics Research Department, NY State Psychiatric Institute; 1994.

30. American-Psychiatric-Association. Diagnostic and Statistical Manual of Mental Disorders. Fourth Edition ed. Washington, D.C.: American Psychiatric Association; 1994.

31. Hamilton M. A rating scale for depression. J Neurol Neurosurg Psychiatry 1960;23:56- 62.

32. Hamilton M. The assessment of anxiety states by ratings. British Journal of Medical Psychology 1959;32:505.

33. Endicott J, Spitzer RL, Fleiss JL, Cohen J. The global assessment scale. A procedure for measuring overall severity of psychiatric disturbance. Arch Gen Psychiatry 1976;33:766-71.

34. Beck AT, Ward CH, Mendelson M, Mock J, Erbaugh J. An inventory for measuring depression. Arch Gen Psychiatry 1961;5:561-71.

35. Ware JE, Jr., Sherbourne CD. The MOS 36-item short-form health survey (SF-36). I. Conceptual framework and item selection. Med Care 1992;30:473-83.

36. Roberts RE. Reliability of the CES-D Scale in different ethnic contexts. Psychiatry Res 1980;2: 125-34.13
